# Locally advanced breast cancer in young women in Latin America

**DOI:** 10.3332/ecancer.2019.894

**Published:** 2019-01-22

**Authors:** Cynthia Villarreal-Garza, Edna A Lopez-Martinez, Jose Felipe Muñoz-Lozano, Karla Unger-Saldaña

**Affiliations:** 1 Breast Cancer Center, TecSalud, Tecnologico de Monterrey, Monterrey 66278, Mexico; 2 Research and Breast Cancer Department, Mexican National Cancer Institute, Mexico City 14080, Mexico; 3 Joven and Fuerte Program for Young Women with Breast Cancer, Mexico City 03720, Mexico; 4 CONACYT fellow—Epidemiology Unit, Mexican National Cancer Institute, Mexico City 14080, Mexico

**Keywords:** young, breast cancer, locally advanced, Latin America, delay

## Abstract

The purpose of this review is to organise, summarise and critically assess existing knowledge on locally advanced breast cancer (LABC) among young women in Latin America. We discuss the most relevant findings in six sections: 1) epidemiology of breast cancer in young women in Latin America; 2) being young as a factor for worse prognosis; 3) LABC in young women in the region; 4) aggressive tumour behaviour among young women; 5) delays in diagnosis and treatment and 6) burden of advanced disease. We point out the need to dedicate resources to enhance earlier diagnosis and prompt referrals of young women with breast cancer; promote research regarding prevalence, biologic characteristics, outcomes and reasons for diagnosis and treatment delays for this age group; and finally, implement supportive care programmes as a means of improving patients and their families’ well-being. The recognition of the current standpoint of breast cancer in young patients across the continent should shed some light on the importance of this pressing matter.

## Introduction

Breast cancer (BC) is the leading cause of cancer-related death and disability among young women in Latin America [1–3]. As 13% of BC deaths occur in women aged < 45 years, this represents a significant burden across the region [4]. Overall, young women with breast cancer (YWBC) are often diagnosed in late disease stages [5, 6] and, thus, undergo more aggressive treatment regimens with considerable morbidity, socio-economic repercussions and poor outcomes [7, 8]. Furthermore, the prognosis of YWBC in Latin America is worse than that in developed countries, mainly due to late disease stage at presentation, especially when coupled with inequities in access to care and inadequate health system capacity for achieving timely diagnosis and treatment [1]. Thus, BC occurring at younger ages represents an extra challenge in terms of prevention, early detection, treatment and survivorship care in this region [9].

The purpose of this review is to organise, summarise and critically assess existing knowledge on locally advanced breast cancer (LABC) among young women in Latin America. The recognition of the current standpoint of BC in young patients across the continent should shed some light on the importance of this pressing matter.

An extensive literature search was conducted in the following electronic databases: PubMed, Cochrane and SciELO. Also, records of previous relevant annual cancer conferences, such as the American Society of Clinical Oncology Annual Meeting, the European Society of Medical Oncology Annual Congress and the San Antonio Breast Cancer Symposium were consulted. The following keywords were used in various combinations, both in English and Spanish: locally advanced breast cancer, LABC, locally advanced disease, stage III, stage IIB, young, < 40 years, < 35 years, < 30 years, epidemiology, delay, time intervals, intervals of care, diagnosis interval, health system interval, health provider interval, Latin America, Latin American, low and middle-income countries (LMICs), LMICs, Central America, South America and the name of each country in Latin America. We included manuscripts or abstracts published between 1992 and 2017. Additionally, reference lists of studies included in this review were hand-searched for relevant publications.

Two reviewers screened the titles and abstracts of all records for relevance and assessed potential ones for inclusion. Disagreements were resolved by discussion with a third reviewer. Studies that reported young women diagnosed with LABC, stage II or stage III BC, or that were primarily aimed at describing or analysing BC in Latin American countries were included in this analysis. One hundred and ninety-nine manuscripts/abstracts were identified through the electronic database search and six through manual searches of references in relevant studies. 158/205 were excluded after reviewing the title and abstract because they did not address the research topics and 47/205 were selected for full-text review. Of these, seven additional papers were excluded because they did not contain relevant information for this research. Forty manuscripts/abstracts were included in the final analysis.

One of the limitations faced during the information search was the scarcity of data addressing LABC in Latin America. Thus, the information included in this critical review comprises results from individual institutions, mainly from Mexico and Brazil, which might compromise the generalisability of the findings. Moreover, due to the non-standard definitions of young and locally advanced disease, manuscripts and abstracts reviewed reported heterogeneous evidence, which made data analysis challenging.

In this critical review, relevant information is presented in the following sections: 1) epidemiology of BC in young women in Latin America; 2) being young as a factor for worse prognosis; 3) LABC in young women in the region; 4) aggressive tumour behaviour among young women; 5) delays in diagnosis and treatment and 6) burden of advanced disease.

## Epidemiology of breast cancer in young women in Latin America

**Figure 1.**
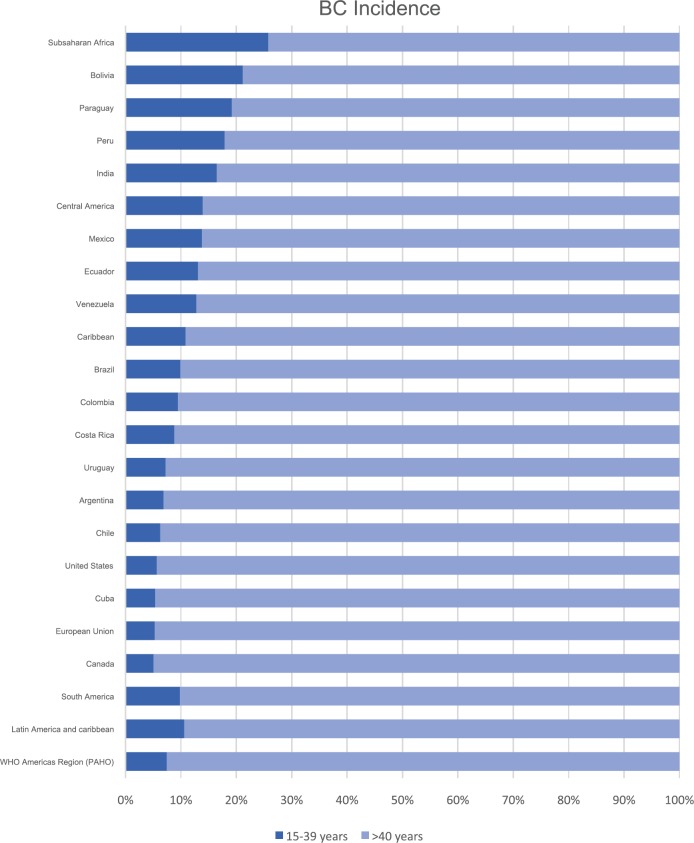
BC incidence by age group (15–39 versus ≥40 years) according to GLOBOCAN 2012 [4].

BC in young women is a significant public health issue, especially in Latin American countries, where the proportion of BC in young patients aged < 40 and < 44 years reaches up to 11% and 20%, respectively, a higher proportion compared to the incidence rates of developed countries such as USA and Canada, which approximates 5% and 11% in each age group (Figure 1) [4]. A possible contributor to this phenomenon is the younger age distribution in Latin American populations [9]. However, it has been reported that up to one-third of the incidence of BC cases in young women might not be explained by the younger age distribution in Latin American countries, as demographic, socioeconomic, genetic and lifestyle-related risk factors could also be important contributors [9].

Several studies in Latin American countries have assessed the prevalence of BC among young patients and found a greater burden of disease than expected. In 2001, a Mexican study reported that the prevalence of patients < 40 years was 16.4% [10]. As for Brazil, a cohort of 59,317 patients reported that the prevalence of patients ages 18–38 years was 10.9% [11]. The Bahamas is another notable example reporting a 12% prevalence of young patients in the year 2011 [12].

Notably, BC is one of the main causes of death in young women < 40 years across Latin America, accounting for 7% of all BC deaths in women [4], with the highest rates seen in Venezuela, Bolivia, Peru and Mexico, with 8%, 9%, 9% and 11%, respectively [4]. Various studies in Latin American countries have addressed this unmet growing healthcare concern. For example, in a descriptive study of mortality due to cancer in women in Sao Paulo, Brazil, the main cause of death for the group aged 30–49 years was BC [13]. Likewise, according to the Mexican population registry Instituto Nacional de Estadistica y Geografia (INEGI), BC is the main cause of cancer death in women aged 20 and older [4, 14]. Furthermore, another study from Brazil has shown an increasing trend in BC mortality in women aged 20–49 years [15].

## Being young as a factor for worse prognosis in Latin America

Studies in Latin American countries have confirmed that being young is an independent risk factor for recurrence and worse survival rates. In a study from Chile that compared survival rates at 5 years between patients ≤40 and ≥70 years old, with stage I to III disease, it was concluded that young patients had a higher recurrence rate than older women (25.8% versus 11.7%) but had lower mortality (17.3% versus 29.4%), potentially due to lower chemotherapy use in the elderly population with high-risk tumours [16]. Similarly, in a historical cohort of women with BC, diagnosed between 2000 and 2002 in Brazil, age < 30 years was significantly associated with increased risk of death (hazard ratio (HR) = 3.09; 95% confidence interval (CI): 1.25−7.67) [17]. Another Mexican study revealed that young patients had poorer disease-free survival (DFS) and overall survival (OS) compared to those > 40 years (75.6% versus 85.7%, *p* < 0.001 and 78.6% versus 83.4%, *p* = 0.012, respectively) [18]. In an observational report from the same group in Mexico, patients with BC undergoing neoadjuvant treatment were evaluated, and while young women achieved higher pathologic complete response rates, the recurrence-free survival interval was shorter for young patients with and without pathologic complete response, compared with their older counterparts [19]. Finally, in a descriptive study of 323 cases of stage III BC treated in a referral hospital in Mexico, in which 60% of patients were < 50 years, 5-year DFS was only 35% [20].

## Locally advanced breast cancer in young women in Latin America

Although there is scarce information addressing this issue and cancer registries in the region are lacking, available data show a high prevalence of advanced disease in Latin America. Table 1 summarises the most relevant data regarding YWBC and LABC in Latin American countries.

Studies from Mexico have shown a prevalence of LABC in young women that ranges from 43.5% to 68%. The largest BC retrospective cohort conducted at the ‘Instituto Nacional de Cancerologia (INCan)’ in Mexico City, including 4315 BC patients, reported a statistically significant higher prevalence of larger tumours, positive lymph node disease and stage III BC in young women ≤ 40 years when compared with older patients (29%, 39.5% and 43.5% versus 17%, 29.9% and 34.9%, respectively). For patients without metastatic disease at diagnosis, 5-year DFS was lower in women ≤ 40 years compared with those > 40 years (75.6% versus 85.7%, *p* < 0.001). This difference was mainly attributable to a significantly lower 5-year DFS in younger women with stage III tumours (*p* = 0.01) [18]. An additional retrospective cohort conducted at INCan that involved 320 YWBC ≤ 42 years old reported that 67.5% of patients were diagnosed with locally advanced disease [21].

Additional studies conducted in Mexico have confirmed the high prevalence of LABC in young women. The previously mentioned study that involved 323 patients diagnosed with stage III BC found that 60% of them were < 50 years [20]. Furthermore, in a retrospective study conducted at the ‘Instituto de Enfermedades de la Mama FUCAM’, a total of 68/142 (48%) young women < 40 years were diagnosed with locally advanced disease. A total of 13 recurrences were documented in a median of 29.5 months, 92% of which were in patients with advanced stages at diagnosis [22]. Finally, results from the first 243 YWBC patients from the prospective cohort ‘Joven & Fuerte’ showed that most patients had stage II (40.3%) or stage III disease (37.9%) [23].

Similar tendencies have been described in Brazil. In a cross-sectional study of 59,317 women using data from Brazilian hospital registries, 63% of young women < 40 years were diagnosed at advanced stages (IIB–IV). In this study, being young was associated with advanced disease at diagnosis, along with having a low level of education and living in the poorest regions [11]. Moreover, a retrospective cohort of 738 patients conducted in Brazil found stage II and stage III to be the most common stages at presentation in young women ≤ 40 (36% and 27%, respectively) [17].

Additionally, two studies from Mexico have reported a high prevalence of genetic mutations and locally advanced disease in young patients with BC. In a prospective cohort of 190 Mexican women < 50 years with triple negative BC, the majority presented with locally advanced disease (69%), median tumour size was 4 cm and a BRCA mutation was detected in 23% of patients [24]. Furthermore, a retrospective study of women < 45 years found that 5/78 (6.4%) had a TP53 mutation. All five patients were < 36 years and all had locally advanced disease [25]. However, advanced presentation at early ages seems to be related not to the mutation status itself but follows the general BC presentation patterns seen in young Latin American women.

**Figure 2.**
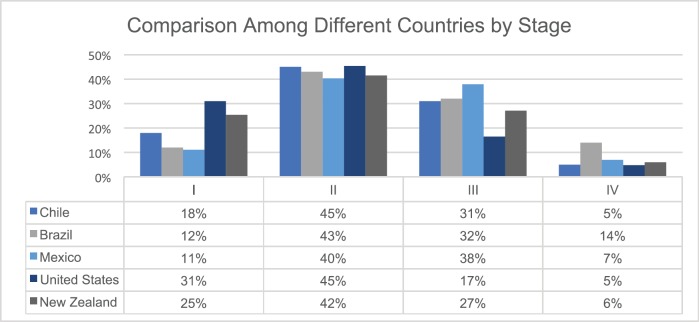
BC stages at diagnosis in selected countries in Latin America [16, 17, 23], USA [26] and New Zealand [27].

To highlight the higher advanced stages at diagnosis in Latin American countries when compared to the developed world, a comparison of stage distribution in Mexico [23], Brazil [17], Chile [16], USA [26] and New Zealand [27] is shown in Figure 2.

There are two possible explanations for younger women presenting with more advanced stage BC: 1) a biologically more aggressive tumour behaviour that leads to faster tumour growth and dissemination and 2) greater diagnosis delays among younger women. These mechanisms will be described in the following sections.

## More aggressive tumour behaviour and LABC among young women

The high proportion of advanced stages at presentation seen in younger patients may be in part due to more aggressive tumour behaviour when compared to their older counterparts, as previously reported by several groups [28, 29]. YWBC tend to have large tumours with lymph node involvement, which is ultimately responsible, at least in part, for the poor prognosis seen in this population [29, 30]. Also, young women have an increased proportion of high-grade tumours and hormone receptor-negative, triple-negative and luminal B tumours, which have higher mortality at regional/distant stages than Luminal A subtypes [31]. Notably, young age seems to be particularly prognostic in women with luminal BC, which accounts for approximately 60% of tumours seen in this population [32]. Additionally, even when adjusted for clinical stage, YWBC have a worse prognosis, with higher rates of systemic relapse and lower OS than older women, which may be a consequence of tumour biology itself [28, 29]. Several studies in Latin America support this claim.

In the previously mentioned 4315-patient retrospective cohort from the INCan in Mexico, tumours in young women were more often high grade (60.9% versus 49.6%, *p* < 0.001), oestrogen receptor-negative (40.5% versus 28.7%, *p* < 0.001) and progesterone receptor-negative (44.1% versus 36.5%, *p* < 0.001) than patients > 40 years. There was also a higher proportion of triple-negative BC in young women (23% versus 14.8%); however, there was no difference in DFS for this subtype between younger and older women. The highest difference in DFS between women ≤ 40 years and older women was found both in Luminal A (H-score above 200) (76.6% versus 88.2%, *p* = 0.04) and Luminal B (H-score under 200) tumours (72.4% versus 86.2%, *p* < 0.001) [18]. On a similar note, a prospective cohort of BC patients undergoing neoadjuvant treatment found that poor survival in young patients could be explained by the worse prognosis in the most prevalent hormone receptor-positive/HER2-negative subgroup, possibly due to a greater proportion of Luminal B tumours, tamoxifen resistance or poor adherence to hormonal treatment [19].

Furthermore, in a Brazilian retrospective cohort of 738 BC patients, the younger group had a higher proportion of multifocal and bilateral cancer when compared with the older group (6.1 versus 2.5%, *p* = 0.017 and 9.8 versus 5.8%, *p* = 0.037, respectively), as well as higher frequency of low degree of differentiation and triple negative tumours (23.1 versus 16.6%, *p* = 0.035 and 10.1 versus 6.4%; *p* = 0.027) [17]. However, this study found no differences in OS rates between groups (*p* = 0.421).

Thus, studies from Latin American countries support the fact that more aggressive tumour characteristics are prevalent among the young BC population, which contributes to the overall burden of advanced disease [1, 7].

## Delays in diagnosis among young women

The other possible explanatory mechanism of the more advanced clinical stages observed among BC in young women is that they face delays in diagnosis. Longer time intervals to initiation of care have been reported to negatively impact clinical stage and survival of BC in the general population [33, 34], probably as a result of late diagnosis and delayed treatment initiation. Studies have shown that as time to care is prolonged, the probability of patients presenting at advanced stages increases [35–37]. This association between delay and survival has been shown to disappear when controlling for clinical stage [38], thus suggesting that delay is not an independent factor for a worse outcome but is linked to the advanced stage at presentation with further BC progression.

Several studies have reported greater delays in the health system interval (time from first medical consultation to treatment start) [39–41] and diagnostic interval (time from the first presentation to healthcare providers to diagnosis confirmation) [42–44] for young BC patients in comparison to their older counterparts. A similar number of studies have not confirmed an association between young age and longer health system [45] and diagnostic interval [6, 46, 47]. However, there are methodological differences between the studies that complicate their comparability.

Among the studies that confirm these associations, they all analysed only symptomatic women, most measured age either as a continuous variable [33, 41, 42] or categorised young age as being < 40 years old [44]. The only one that defined age as < 50 had a very large sample size (>9000) [39]. Therefore, the evidence available seems to favour the relationship between young age and delayed diagnosis.

As for the reviews that reported no association between young age and delay, two of them found significant crude associations that then disappeared when controlling by symptom presentation [6, 47], menstrual status [47] and history of benign breast conditions [47], which are all related with young age. The remaining negative studies defined young age as < 50 years, had a small sample size (n = 380) or focused on women identified through screening mammography [48], which could be the reasons they did not find a significant association between age and delay. It could also be that this relationship is different in diverse health system contexts.

On the other hand, only three quality studies have analysed the effect of young age on the treatment interval (time between diagnosis confirmation and cancer treatment initiation) and also showed contradictory results. One found an increased risk of delayed treatment among younger women [49], another reported a decreased risk in this group [46] and the last one found no association [48].

There are very few studies reporting BC time intervals of care in Latin America and even fewer that focus on the health system or diagnostic intervals [50–54]. Table 2 summarizes findings and main characteristics of these studies. Most studies report very long diagnostic intervals, with the shortest median reported at 3 months in the Colombian study [50]. These delays are most likely a consequence of the inequitable availability and quality of cancer services, including healthcare personnel, infrastructure and diagnostic equipment, which make access to cancer care across Latin American countries challenging [55].

Unfortunately, none of the Latin American studies of time intervals for BC care focus on the relationship between age and the length of the health system or diagnostic intervals. Nevertheless, several studies included age in their multivariate analyses but did not find significant associations. However, it is interesting to note that one study identified mechanisms that explain diagnosis delay among BC patients in Mexico City [56]. In this report, perceived medical errors in primary care services were significantly associated with longer diagnostic intervals and young age, as well as symptomatic presentation. Furthermore, a recent study that evaluated BC knowledge among general physicians in Mexico found very low levels of knowledge about screening recommendations [57].

In conclusion, although evidence that supports an association between age and diagnosis delay is scarce, it can be hypothesised that young age conveys a higher risk of diagnosis delay due to: 1) a greater difficulty for both patients and physicians to suspect BC in this low cancer-risk group, 2) the common occurrence of benign breast conditions in younger women and 3) the fact that screening mammography is not useful for women < 40 years of age.

## Burden of advanced disease in young women

Burden of disease derives not only from the high morbidity and mortality rates encountered in YWBC, but also on the profound and lasting effects on self-development, family dynamics, social and professional lives [7, 58]. Particularly challenging age-related issues associated with early and long-term morbidity include: chemotherapy-induced premature ovarian failure, infertility, body image disturbance and compromised sexual function, among others [7, 8]. As a notable example, initial results from the Mexican ‘Joven & Fuerte’ cohort of YWBC showed significant morbidity at the time of diagnosis and worsening at 6-month follow-up. Mexican women have shown high rates of sexual dysfunction at baseline and 6 months (61.4% and 74.3%, respectively, *p* < 0.001), as well as an elevated proportion of low sexual satisfaction at both moments (40.6% and 43.5%, *p* = 0.004) [23]. Furthermore, Mexican YWBC experience significant deterioration in many quality of life domains during the first year of follow-up, with a marked decrease at 6 months [59]. Conversely, emotional functioning and future perspective improved over time [59].

Moreover, in Latin American countries, where there is a predominant lack of financial resources across the region, YWBC face extra challenges to access supportive medical interventions that are not routinely covered. For example, in a cross-sectional study involving 134 newly diagnosed young Mexican BC patients, although 48% reported a willingness to have children prior to BC diagnosis, only 3% considered that they could afford extra expenses [23]. In comparison, 50% to 60% of young BC patients in the USA feel financially comfortable/can afford ‘special things’ [60, 61].

Additionally, BC has a strong economic impact, especially in young patients, as the majority of women < 54 years are economically active [62] and prolonged sick leave can cause financial difficulties and emotional distress [63]. An observational study in a third-level hospital in Brazil evaluating return to work rates in BC patients 18–57 years old found that although 61.5% received support from their employers, only 29.1% reported adjustment offering so that they could keep working during treatment [63]. Overall, 22.1% and 28.8% of patients had returned to work at 6 and 12 months after BC diagnosis, respectively.

The burden of BC can be objectively described in terms of disability-adjusted life years (DALYs), a measure that combines years of potential life lost to premature mortality and years of productive life lost due to disability. Overall, a total of 613,000 DALYs are lost in Latin American countries due to BC [64]. Although the general social burden is lower than in developed countries, where incidence rates of BC are highest, the proportion of losses due to mortality is greater in Latin America as a consequence of higher mortality rates and younger age of women at diagnosis [64].

Young age at diagnosis and death entails a heavy burden in countries like Brazil, Peru and Mexico [64]. In Brazil, for example, DALYs lost per 100,000 women are nearly double compared to most countries in the region, which might be explained by the increasing BC incidence and mortality in young women < 40 years, as well as, an increasing number of diagnoses in working-age women which greatly contributes to lost years. Likewise, in Mexico, significant productivity losses occur due to the young age at diagnosis and death.

## Key intervention strategies

Due to the high prevalence of LABC in young women in Latin America and the burden of disease, this issue deserves significant attention since its diagnosis requires high clinical suspicion [17]. Because data in Latin America has shown that 90% of BCs in young women are self-detected [65] and routine mammography screening is not recommended for this age group, it is imperative to dedicate resources for general public education about the possibility of a malignant diagnosis in young women. Also, healthcare providers should be sensitive to the current YWBC standpoint and not automatically discard this diagnostic possibility because of young age. Furthermore, it is equally important to educate patients and caregivers about the presence of hereditary cancer risk factors and promptly refer high-risk patients to genetic counselling. If young mutation carriers were regularly identified, timely preventive measures could be implemented and earlier detection of BC achieved.

Early diagnosis strategies could be especially useful among young women living in Latin America, which comprises mainly low and middle-income countries that lack population-based BC screening programmes. These strategies should include interventions directed to: 1) improve BC awareness among the population at risk, particularly of red flag breast symptoms, relevance of prompt medical attention seeking and available health services; 2) strengthen local health systems capacity to achieve a prompt and accurate diagnosis (e.g. primary care, breast imaging diagnostic services, pathology facilities) and 3) improve timely access to quality treatment (e.g. making treatment accessible without cost for the most vulnerable, developing shorter referral routes).

Examples of early diagnosis strategies that are currently underway are encouraging. Preliminary findings of a randomised controlled study from the Cairo Breast Screening trial demonstrated that screening based on breast self-examination combined with clinical evaluation resulted in downstaging of tumours in the intervention arm compared with the control arm [66]. Remarkably, the Mexican navigation programme ‘Alerta Rosa’, which focused in prioritising and navigating symptomatic patients and those with abnormal breast findings for prompt diagnosis and treatment initiation, proved effective in reducing health system delays, with a median of 33 days from the first contact to treatment initiation [67]. This programme is currently ongoing, with the ultimate goal of BC downstaging, thorough its gradual implementation across the country [68].

Finally, as YWBC have an added load of unique concerns, comprehensive programmes have been created worldwide, predominantly in the USA and Canada [7, 8, 69]. However, in limited-resource settings, cancer-control efforts are predominantly aimed at improving medical care, while supportive care and survivorship issues are not highly prioritised and often neglected. Considerable morbidity due to advanced disease and aggressive treatment, coupled with a lack of support services and financial limitations, greatly undermine patients’ quality of life in Latin American countries [1]. Thus, models of supportive care should be implemented for this particular group of patients to provide better care for this emergent challenge. In 2014, the first dedicated programme for the care of young BC patients in Latin America was implemented. ‘Joven & Fuerte: Programme for Young Women with Breast Cancer in Mexico’ aims to optimise young patients’ clinical and psychosocial care, enhance education regarding their special needs and promote targeted research [23]. To date, this programme has provided care for up to 500 patients and seeks to promote its replication in other health care centres across Mexico and Latin America.

## Conclusion

The data presented urges Latin American countries to consider dedicating resources to enhance earlier diagnosis and prompt referrals of YWBC. Special efforts are needed for younger women, as mammography screening is not recommended in this population. Additionally, more research is required in Latin America regarding the prevalence and outcomes of BC in young women, its biological characteristics and reasons for diagnosis and treatment delays. Finally, supportive care programmes for YWBC should be implemented, as a means of improving patients’ and their families’ well-being.

## Conflicts of interest

The authors certify that they have no affiliations with or involvement in any organisation or entity with any financial interest or non-financial interest in the subject matter or materials discussed in this manuscript.

## Funding

The authors received no specific funding for this work.

## Figures and Tables

**Table 1.** Studies addressing YWBC and LABC in Latin American countries.

Country	Reference	Year	Main Focus	Number of patients	Conclusions
Bahamas	K Mungre, *et al* [12]	2016	Retrospective observational sociodemographic description of BC in the Bahamas.	270	The incidence of BC in women < 40 years in the Bahamas was 9% in the year 2009, 8% in 2010 and 12% in 2012. 95.5% of young patients presented with ≥ stage II BC.
Brazil	Abrahao Kde, *et al* [11]	2015	Retrospective observational study to analyse the determinants of advanced stages in Brazilian women with BC.	59,317	63% of young women < 40 years were diagnosed at advanced stages (IIB–IV). Younger age (18 to 49 years old) (odds ratio (OR) = 1.61 95% CI 1.51 to 1.72), having low educational level (OR = 1.53 95% CI 1.48 to 1.58), living in less developed geographical regions (OR = 1.27 95% CI 1.21 to 1.33), having invasive ductal carcinoma (OR = 2.70 95% CI 2.56 to 2.84) and invasive lobular carcinoma (OR = 2.63 95% CI 2.42 to 2.86) were associated with advanced BC.
Brazil	De Lima Vazquez, *et al* [17]	2016	Retrospective, observational study that compares socio-demographic, clinical and pathological characteristics and their association with long-term survival between two random cohorts of young (≤40 years) and older (50–69 years) Brazilian patients with BC.	1,735	Among young women (*n* = 469), a prevalence of 12% in stage I, 43% in stage II, 32% in stage III and 14% in stage IV was reported. The OS rates of the two age groups were similar except when analysed according to treatment period (1997–2002). Although patients aged ≤ 40 years harboured tumours with more aggressive clinicopathological characteristics, these were not independent predictors of OS.
Brazil	Rocha-Brischillari, *et al* [15]	2017	Retrospective, observational study that analyses time trends in overall mortality from BC in Brazil, Brazilian regions and states.	13,870	New cases of BC diagnosed in advanced stages continue to emerge in younger women. Trend: increased mortality in all regions of Brazil in women 20–49 years.
Brazil	Schneider IJ [70]	2009	Retrospective study to analyse BC survival and associated factors based on a historical cohort of women with BC diagnosis from 2000 to 2002.	1008	Overall 5-year survival was 76.2% (95% CI: 73.6–78.9). Independent factors associated with increased risk of death were age < 30 years (HR = 3.09; 95% CI: 1.25–7.67); illiteracy (HR = 3.70; 95% CI: 1.44–9.55) and stages III (HR = 5.27; 95% CI: 2.56–10.82) and IV (HR = 14.07; 95% CI: 6.81–29.06). Young women had the worst survival rates.
Chile	Acevedo Francisco, et al [16]	2015	Retrospective study comparing BC in young patients and the elderly.	2023	Incidence between ≤ 40 years and ≥ 70 years was, respectively: Stage I 18.8% versus 32.2%, Stage II 45% versus 40.1%, Stage III 31.3% versus 19.9% and Stage IV 5% versus 7.8%. The younger cohort had a higher incidence of triple negative (17.8% versus 11.7%) and Luminal B (43% versus 33.3%) BC. Young patients had a higher incidence of recurrence compared to the elderly (25.8 versus 11.7%).
Latin America	Villarreal-Garza, *et al* [1]	2013	Systemic literature review of BC incidence and mortality among young women using data from the Globocan registry, and related clinical, pathological and survivorship aspects in this region.	Not available	Incidence and mortality in women < 45 years in Latin American countries was 20% and 14% versus 12% and 7% in developed countries, respectively. Stage II and III disease, high histological grade and triple-negative and HER2 BC were features frequently observed among young Latin American BC patients.
Mexico	Villarreal-Garza C, *et al* [23]	2017	Initial results of prospective YWBC cohort.	243	98 patients were diagnosed at stage II (40.3%) and 92 at stage III (37.9%). Nine patients (4%) had developed distant recurrences and 12 patients (5%) had died as a consequence of BC, with a median follow-up of 17 months.
Mexico	Robles Castillo, *et al* [22]	2011	Retrospective study that determined the frequency, sociodemographic, clinical and histopathological features of BC in women under 40 years attending a specialist breast unit in Mexico City.	142	45.7% were diagnosed with the early disease (I and IIA), 47.89% of cases with locally advanced disease (IIB – IIIC) and 9% with metastatic disease (IV). A total of 13 recurrences were documented; 92% were in patients with locally advanced disease.
Mexico	Villarreal Garza, *et al* [21]	2013	Retrospective study that described the frequency of BC among young Mexican patients, as well as their pathological characteristics at diagnosis and patterns of recurrence.	320	67.5% diagnosed with locally advanced BC. From the non-metastatic patients at diagnosis, 31% developed recurrence (65% systemic, 21% loco regional and 14% both). After a median follow-up of 26 months, 18% of the 320 patients died secondary to BC disease progression.
Mexico	Villarreal-Garza, *et al* [19]	2015	Retrospective study that compared the recurrence-free survival (RFS) among neoadjuvant-treated patients according to age and histologic subtypes.	3,110	Young patients achieved higher pathologic complete response (PCR) rates (37% versus 25%), but the RFS interval was shorter at the expense of the hormone receptor-positive/HER2-negative subgroup. For patients with residual disease, young age remained a significant independent predictor of recurrence in patients with hormone receptor-positive/HER2-negative tumours but not in the HER2 positive and triple negative subtypes.
Mexico	Villarreal-Garza, et al [18]	2017	Retrospective study of a single institution comparing clinical characteristics, treatment and survival between women ≤ 40 and > 40 years of age. Also, survival analyses were performed for each molecular subtype.	4315	A total of 662 women (15.3%) were ≤ 40 years old. Among young women, 7.6% were diagnosed with stage I, 33.1% with stage II, 43.5% with stage III and 14.5 with stage IV. Younger women had more advanced disease, higher grade and a larger proportion of luminal B and triple-negative tumours (P < .001). At 5 years, both DFS and OS were lower in younger women, although there were no differences after adjusting for stage. Luminal B tumours showed a worse 5-year OS in younger women (79.1% versus 85.2%; P = .03).
Peru	Weibin Lian [71]	2017	Descriptive, retrospective study that aims to compare clinicopathological and outcome characteristics according to patient age at diagnosis and menopausal status.	1024	Stage III was higher in the pre versus postmenopausal group (33.7% versus 26.8%). Premenopausal women had a lower incidence of stage II disease (48.7% versus 57.6%).

**Table 2.** Health system intervals reported for Latin American countries.

Reference	n	Median patient age	Health services interval measured	Median interval	% LABC patients
**Brazil**					
Rezende, 2009 [52]	104	54	Diagnostic interval	6.5 mo	37.5% II & III
Barros, 2013 [54]	250	52	Health system interval	5.2 mo	74.5% II & III
**Colombia**					
Piñeros, 2011 [50]	1106	53.1	Diagnostic interval	3 mo	45% LABC
			Health system interval	4.6 mo	
**Mexico**					
Bright, 2011 [53]	32	53	Diagnostic interval	6.6 mo	70% LABC
Unger-Saldaña, 2015 [37]	597	51	Diagnostic interval	4.2 mo	67% II & III
Angeles-Llerenas, 2016 [51]	854	52	Mammography result to treatment	2.2 mo	79.8% II & III
